# SARS-CoV-2 Point-of-Care Testing Modalities: Integrating Molecular, Immunological, Biosensor, and AI Approaches

**DOI:** 10.3390/diagnostics16152402

**Published:** 2026-07-30

**Authors:** Helal F. Hetta, Rehab Ahmed, Abdul Haseeb, Salwa Qasim Bukhari, Zinab Alatawi, Ahmad J. Mahrous, Mahmoud E. Elrggal, Mohammad Al Masri, Ahmed A. Kotb

**Affiliations:** 1 Division of Microbiology, Immunology and Biotechnology, Department of Natural Products and Alternative Medicine, Faculty of Pharmacy, University of Tabuk, Tabuk 71491, Saudi Arabia; reahmed@ut.edu.sa; 2 Department of Pharmacy Practice, Faculty of Pharmacy, University of Tabuk, Tabuk 71491, Saudi Arabia; ahanif@ut.edu.sa; 3 Department of Diagnostic Radiology, Faculty of Medicine, University of Tabuk, Tabuk 71491, Saudi Arabia; s.bukhari@ut.edu.sa; 4 Department of Family and Community Medicine, Faculty of Medicine, University of Tabuk, Tabuk 47512, Saudi Arabia; zalatawi@ut.edu.sa; 5 Department of Pharmaceutical Practices, College of Pharmacy, Umm Al-Qura University, Makkah 21955, Saudi Arabia; ajmahrous@uqu.edu.sa; 6 College of Medicine, Al Qunfudah Umm Al-Qura University, Al Qunfudhah 28821, Saudi Arabia; merggal@uqu.edu.sa; 7 Faculty of Allied Medical Sciences, Hourani Center for Applied Scientific Research, Al-Ahliyya Amman University, Amman 19111, Jordan; masri@ammanu.edu.jo; 8 Department of Microbiology and Immunology, Faculty of Pharmacy, Assiut University, Assiut 71515, Egypt; ahmedabedelaziz994@aun.edu.eg

**Keywords:** COVID-19, SARS-CoV-2, point-of-care testing, molecular diagnostics, antigen testing, biosensors, artificial intelligence, CRISPR, LAMP, infectious disease surveillance

## Abstract

The coronavirus disease 2019 (COVID-19) pandemic highlighted the critical need for rapid, accessible, and accurate diagnostic tools to support timely clinical decision-making, outbreak control, and public health surveillance. Point-of-care testing (POCT) has emerged as an essential component of decentralized healthcare by enabling diagnostic testing outside conventional laboratory settings. This review was based on a structured literature search of PubMed, Scopus, and Web of Science databases covering studies published between January 2020 and January 2026. The review evaluates current advances in COVID-19 POCT technologies, including molecular assays, antigen-based tests, antibody-based assays, biosensor platforms, and artificial intelligence (AI)-assisted diagnostic approaches. Molecular POCT methods, including rapid reverse transcription polymerase chain reaction (RT-PCR), loop-mediated isothermal amplification (LAMP), and CRISPR-based technologies, provide high analytical sensitivity and specificity and are increasingly suitable for decentralized diagnostic applications. Antigen-based assays offer rapid and cost-effective screening solutions, although diagnostic performance may vary depending on viral load, symptom onset, and circulating variants. Antibody-based POCT remains valuable for seroprevalence studies, retrospective diagnosis, and immune-response monitoring rather than acute infection detection. Emerging biosensor technologies and AI-enabled diagnostic systems demonstrate promising analytical capabilities and operational advantages; however, many remain at the prototype or early-validation stage and require further clinical evaluation before widespread implementation. The findings indicate that no single POCT modality is optimal for all clinical scenarios. Instead, molecular, antigen, antibody, biosensor, and AI-assisted approaches provide complementary strengths that support different diagnostic and public health objectives. Continued advances in assay design, digital connectivity, multiplex testing, and variant-resilient detection strategies are expected to further enhance the role of POCT in COVID-19 management and future infectious disease preparedness.

## 1. Introduction

An outbreak of pneumonia that began in December 2019 in Wuhan, China, was linked to a novel strain of the coronavirus that was provisionally termed the 2019 novel coronavirus by the WHO. Then its name changed to the Severe Acute Respiratory Syndrome Coronavirus 2 (SARS-CoV-2) on 11 February 2020 [1]. The viral ailment was formally identified by the WHO as the Coronavirus Disease 2019 (COVID-19). The symptoms of this illness include respiratory difficulties, fevers, coughing, lethargy, pneumonia, and muscle pain [2,3,4].

On 30 January 2020, the WHO designated the viral epidemic an international public health emergency due to the rise in the number of infected individuals in China. The evolving SARS-CoV-2 is an enveloped, single-stranded, positive-sense RNA virus that belongs to the family Coronaviridae’s Betacoronavirus genus [2,5,6]. The main determinants of viral virulence and function are the nucleocapsid protein (N), membrane glycoprotein (M), and spike glycoprotein (S) [7].

Since the emergence of the Omicron (B.1.1.529) variant in late 2021, the epidemiological landscape of SARS-CoV-2 has undergone continuous evolution. Successive Omicron sub-lineages, including BA.2, BA.4/5, XBB, and subsequently JN.1, have displaced earlier circulating variants through enhanced transmissibility and immune escape. Genomic surveillance conducted in the United States demonstrated the sequential replacement of XBB lineages by JN.1 between 2023 and 2024, reflecting ongoing viral adaptation and selective pressure on circulating strains. More recently, descendants of JN.1, including KP.2 (FLiRT), KP.3 (FLuQE), XEC, and other emerging variants, have continued to dominate global transmission, highlighting the dynamic evolutionary trajectory of SARS-CoV-2 and emphasizing the need for continuous reassessment of point-of-care testing (POCT) performance to ensure reliable detection of currently circulating variants [8,9,10].

Although the acute phase of the COVID-19 pandemic has subsided, SARS-CoV-2 remains an important cause of respiratory illness and continues to circulate alongside seasonal pathogens such as influenza viruses and respiratory syncytial virus (RSV). Consequently, the role of point-of-care testing (POCT) has evolved from supporting emergency outbreak control and mass screening toward facilitating long-term clinical management, integrated respiratory pathogen surveillance, decentralized healthcare delivery, and preparedness for future outbreaks. Contemporary POCT platforms are therefore expected not only to provide rapid and accurate diagnosis but also to demonstrate resilience against emerging viral variants, support multiplex detection of co-circulating respiratory pathogens, enable digital connectivity for public health surveillance, and remain accessible across diverse healthcare settings. These evolving clinical and public health requirements have reshaped the development and evaluation of SARS-CoV-2 POCT technologies beyond the immediate pandemic response [11,12].

The current SARS-CoV-2 virus produces lower respiratory tract infections and could result in acute respiratory distress syndromes, much like the previous epidemic of coronavirus. SARS-CoV-2 RT-PCR testing of clinical specimens, such as nasopharyngeal swabs and sputum, is now the main diagnostic technique for SARS-CoV-2 infection. Other techniques are also in use, including electrochemical biosensors, lateral flow immunochromatographic assays, isothermal (nucleic acid amplification testing) NAAT and AI-assisted diagnostics.

The majority of the diagnostic assays utilized today require large, automated devices because most testing is done at medical centers and independent testing facilities. Point-of-care diagnostics are helpful in light of its simplicity, increased user friendliness, early identification, and equivalent accuracy and sensitivity, which could lessen the testing burden on central facilities. As a result, POCT helps with everyday outbreak control as well as early diagnosis and management [13,14,15,16,17].

This review provides a comprehensive overview of current point-of-care testing (POCT) technologies for SARS-CoV-2. In this context, POCT encompasses decentralized or near-patient diagnostic platforms that operate outside centralized laboratory settings, including portable and home-based systems. The present review is framed within the post-pandemic phase of COVID-19 (2025–2026), a period characterized by a transition from emergency outbreak response to sustainable surveillance, decentralized healthcare delivery, and preparedness for future respiratory disease threats. Although large-scale laboratory testing was essential during the acute phase of the pandemic, contemporary diagnostic strategies increasingly prioritize rapid, accessible, and cost-effective POCT solutions for community, home-based, and resource-limited settings. Moreover, the continued evolution of SARS-CoV-2 variants and the growing demand for integrated respiratory pathogen surveillance underscore the importance of adaptable diagnostic platforms capable of maintaining performance under changing epidemiological conditions.

## 2. Methods for Literature Review and Evidence Selection

A structured narrative review was conducted to identify peer-reviewed studies on point-of-care testing (POCT) technologies for SARS-CoV-2 detection, encompassing molecular, immunological, biosensor-based, and AI-assisted diagnostic approaches. Literature searches were performed in PubMed, Scopus, and Web of Science for studies published between January 2020 and January 2026.

Search strategies combined controlled vocabulary and free-text terms, including “SARS-CoV-2”, “COVID-19”, “point-of-care testing”, “rapid diagnostics”, “molecular assays”, “biosensors”, “lateral flow immunoassay”, “isothermal amplification”, “CRISPR diagnostics”, and “artificial intelligence”, using Boolean operators (“AND” and “OR”). Reference lists of included articles were additionally screened manually to identify further relevant studies.

Studies were eligible for inclusion if they described POCT or near-patient diagnostic technologies for SARS-CoV-2 and reported analytical or clinical performance characteristics. For emerging biosensor and AI-assisted technologies, proof-of-concept and laboratory prototype studies were also included due to the limited availability of clinically validated data in these rapidly evolving fields. Exclusion criteria included editorials, conference abstracts, duplicate publications, non-peer-reviewed sources, and studies not focused on SARS-CoV-2 diagnostic applications or lacking sufficient methodological information for extraction.

Data extraction encompassed diagnostic principle, assay format, reported analytical performance (sensitivity, specificity, limit of detection), sample type, regulatory approval status, and intended deployment context. Due to substantial heterogeneity in study designs, diagnostic platforms, and reported performance metrics, extracted information was synthesized narratively rather than through formal meta-analysis.

It is acknowledged that the included technologies represent different stages of development and clinical validation. Established commercial platforms—such as cartridge-based molecular systems and lateral flow antigen assays—have undergone extensive clinical evaluation and regulatory review, whereas several emerging technologies remain at the laboratory prototype or pilot validation stage. Biosensor-based and AI-assisted platforms are included to highlight emerging technological directions and translational potential, rather than to imply equivalent clinical readiness across all reviewed modalities. Where studies report only experimental or proof-of-concept findings, this is noted explicitly within the relevant sections and tables to allow appropriate contextualization of the evidence.

Commercial assays included in this review were selected to provide representative examples of widely used point-of-care technologies across the principal diagnostic categories (molecular, antigen, antibody, and biosensor-based platforms). Selection was based on one or more of the following criteria: (i) authorization by major regulatory agencies, including the U.S. Food and Drug Administration (FDA) under Emergency Use Authorization (EUA) or 510(k), CE marking, or other recognized national regulatory authorities; (ii) availability of peer-reviewed clinical performance data; (iii) widespread clinical implementation or commercial availability; and (iv) representation of different technological principles. The tables are intended to provide illustrative rather than exhaustive comparisons and therefore do not include every commercially available SARS-CoV-2 POCT platform.

## 3. Point-of-Care Testing

**Figure 1:**
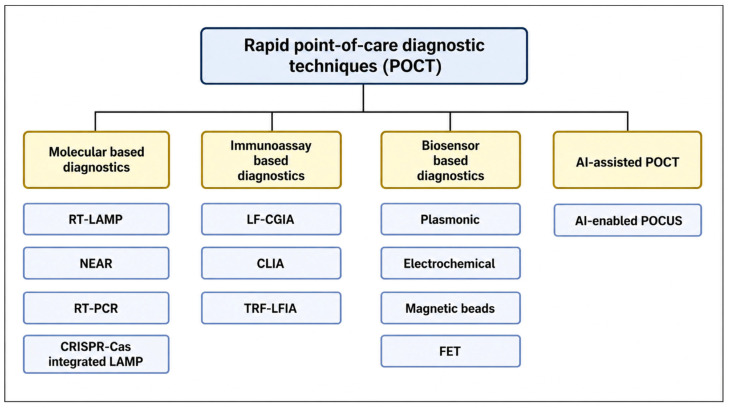
Classification of rapid point-of-care diagnostic techniques. These methods are divided into four main categories: (1) Molecular-based diagnostics, including Reverse Transcription Loop-Mediated Isothermal Amplification (RT-LAMP), Nicking Enzyme Amplification Reaction (NEAR), Reverse Transcription Polymerase Chain Reaction (RT-PCR), and CRISPR-Cas integrated Loop-Mediated Isothermal Amplification (CRISPR-Cas integrated LAMP); (2) Immunoassay-based diagnostics, including Lateral Flow Colloidal Gold Immunoassay (LF-CGIA), Chemiluminescent Immunoassay (CLIA), and Time-Resolved Fluorescence Lateral Flow Immunoassay (TRF-LFIA); (3) Biosensor-based diagnostics, including plasmonic, electrochemical, magnetic beads, and Field-Effect Transistor (FET) technologies; and (4) Artificial Intelligence (AI)-assisted diagnostics such as AI-assisted Point-of-Care Ultrasound (AI-POCUS). Each category employs specific technologies to provide rapid, accurate, and accessible diagnostic solutions. Created in BioRender. Hetta, H. (2026) https://BioRender.com/bu64h2i (accessed on 10 June 2026).

Point-of-care testing (POCT) for the identification of SARS-CoV-2 can be broadly categorized into molecular-based assays, antigen-based tests, antibody-based assays, and emerging technologies such as biosensor platforms and artificial intelligence (AI)-assisted diagnostics (Figure 1). These approaches differ substantially in their detection principles, analytical sensitivity, turnaround time, and operational requirements.

Unlike conventional laboratory diagnostics that often require extended processing times and centralized facilities, POCT enables rapid, decentralized testing. This capability has transformed healthcare delivery by allowing communities, workplaces, and educational institutions to perform timely screening in a practical and cost-effective manner. POCT is particularly valuable in settings where molecular diagnostics such as PCR are unavailable or where rapid decision-making is essential [18,19,20,21,22,23].

Molecular assays detect viral genetic material and generally provide the highest analytical sensitivity, especially during early infection. Antigen-based tests detect viral proteins and offer faster, more affordable screening options suitable for large-scale use. In contrast, antibody-based assays detect host immune responses and are primarily useful for retrospective assessment and epidemiological surveillance rather than early diagnosis [21,23,24]. Emerging biosensor and AI-based technologies aim to further enhance speed, sensitivity, and automation, although many of these platforms are still undergoing clinical validation [25,26,27]. Importantly, these diagnostic modalities should not be viewed as interchangeable. Instead, they function as complementary tools, each suited to specific clinical contexts, stages of infection, and resource environments. Understanding their relative strengths and limitations is therefore essential for optimizing SARS-CoV-2 diagnostic strategies in decentralized healthcare settings [20,23].

### 3.1. Molecular POCT

Laboratory-based nucleic acid testing for SARS-CoV-2 involves a multistep workflow that begins with specimen collection from the upper respiratory tract, followed by RNA extraction, reverse transcription into complementary DNA (cDNA), and nucleic acid amplification using techniques such as reverse transcription polymerase chain reaction (RT-PCR) or loop-mediated isothermal amplification (LAMP). These processes are traditionally conducted in centralized laboratories and require specialized instrumentation, controlled environments, and trained personnel. To address these limitations, point-of-care molecular testing has emerged as an alternative approach aimed at integrating and simplifying the entire workflow into automated systems that can be deployed at or near the patient site without reliance on full laboratory infrastructure [13,28].

The COVID-19 pandemic significantly accelerated the development and deployment of decentralized molecular diagnostic platforms. This led to the emergence of fully integrated systems designed for near-patient and home-use applications, with the goal of approaching laboratory-level performance in a simplified format. These platforms commonly employ cartridge-based automation, rapid real-time PCR, or isothermal amplification strategies to reduce turnaround time while maintaining diagnostic sensitivity. Through integration of sample processing, amplification, and detection within closed systems, these technologies represent a shift toward more accessible molecular diagnostics beyond conventional laboratory settings [29,30,31].

Commercial molecular point-of-care platforms (Table 1) include both cartridge-based RT-PCR systems and isothermal amplification assays, many of which have demonstrated good agreement with standard laboratory PCR methods in specific clinical evaluation studies. By integrating sample preparation, nucleic acid amplification, and detection into a single automated device, these systems enable reliable testing in decentralized and near-patient environments. However, their performance may still be influenced by factors such as viral load, specimen quality, and assay design.

Despite their strong analytical performance, molecular POCT systems are not without limitations. They often require proprietary instrumentation, involve higher operational costs, and generally offer lower throughput compared with centralized laboratory testing. In addition, while ultra-rapid amplification methods improve turnaround time, they may exhibit reduced sensitivity in samples with low viral load, reflecting an inherent trade-off between speed and analytical performance [30,32].

While isothermal amplification methods such as LAMP and NEAR have improved the speed of molecular diagnostics, CRISPR-based systems offer an additional layer of specificity through programmable nucleic acid recognition. These methods utilize Class 2 CRISPR-associated (Cas) enzymes, including Cas9, Cas12, and Cas13, which exhibit sequence-specific recognition combined with nuclease activity. While Cas9 and Cas12 primarily target double-stranded DNA, Cas13 is RNA-guided and specifically targets single-stranded RNA.

A distinguishing feature of Cas12 and Cas13 enzymes is their collateral cleavage activity, whereby target recognition triggers nonspecific cleavage of surrounding nucleic acid sequences. This property is exploited in diagnostic platforms by incorporating reporter molecules labeled with fluorophores; upon activation, cleavage of these reporters generates detectable signals, enabling rapid identification of viral genetic material.

Several CRISPR-based diagnostic platforms have been developed for SARS-CoV-2 detection, including Specific High-sensitivity Enzymatic Reporter Unlocking (SHERLOCK), DNA Endonuclease-Targeted CRISPR Trans Reporter (DETECTR), Cas3-Operated Nucleic Acid detection (CONAN), and Variant Nucleotide Guard (VaNGUARD). These systems are typically combined with isothermal amplification techniques to enhance sensitivity and reduce detection time.

CRISPR-based diagnostics combine the high specificity of nucleic acid recognition with rapid signal generation, offering a potential alternative to conventional molecular assays. Compared to RT-PCR-based POCT, these systems may provide shorter analytical turnaround times with reduced instrumentation requirements. However, despite promising analytical performance, most CRISPR-based platforms remain in early stages of clinical validation, with challenges related to standardization, scalability, and regulatory approval limiting their widespread implementation in routine diagnostic settings [33,34,35,36,37].

Although many POCT platforms have reported high sensitivity and specificity under controlled laboratory conditions, their performance in real-world settings may differ significantly. A large systematic review including 123 studies reported a pooled sensitivity of approximately 92.8% for molecular POCT and 70.6% for antigen-based POCT, with substantial variability depending on clinical context and testing conditions [17].

Antigen-based assays show reduced sensitivity in asymptomatic individuals and when viral load is low, with pooled estimates around 70–75% sensitivity in real-world studies. These findings highlight the importance of considering patient characteristics, sampling technique, and timing of testing when interpreting POCT results [21,38].

The emergence of SARS-CoV-2 variants has raised concerns regarding the reliability of diagnostic assays. Several studies have demonstrated that mutations associated with variants of concern, particularly Omicron, can affect test performance. For example, real-world clinical evaluations have shown reduced sensitivity of antigen-based assays for Omicron compared to earlier variants, with reported sensitivity decreases linked to viral load and assay detection limits. Similarly, comparative analyses have confirmed that rapid antigen tests exhibit lower sensitivity for Omicron relative to Delta, especially in samples with lower viral concentrations. In contrast, molecular assays generally maintain higher robustness due to targeting conserved genomic regions; however, variability in performance has still been observed depending on assay design and target selection. Large-scale reviews have highlighted significant heterogeneity in diagnostic accuracy across different platforms and settings, emphasizing the need for continuous monitoring and reassessment of test performance as new variants emerge [21,39,40].

The continuous emergence of SARS-CoV-2 variants has necessitated ongoing optimization of molecular assay design, particularly with respect to primer and probe selection. Early in the pandemic, several RT-PCR assays incorporated the spike (S) gene as one of their diagnostic targets. However, the Δ69–70 deletion present in the Alpha variant and subsequently in several Omicron lineages caused S-gene target failure (SGTF) in assays such as the Thermo Fisher TaqPath COVID-19 test, whereby amplification of the S gene failed despite successful detection of other viral targets. Although SGTF proved useful as an epidemiological marker for variant surveillance, it highlighted the vulnerability of assays targeting highly variable genomic regions. Consequently, contemporary molecular POCT platforms have increasingly adopted multiplex primer/probe designs directed against more conserved regions of the viral genome, particularly the nucleocapsid (N), membrane (M), envelope (E), ORF1ab, and RNA-dependent RNA polymerase (RdRp) genes. These conserved targets exhibit lower mutation frequencies than the S gene, thereby reducing the likelihood of diagnostic escape while maintaining analytical sensitivity across emerging Omicron sub-lineages. The use of multiple conserved genomic targets also provides redundancy, minimizing false-negative results caused by mutations affecting a single primer or probe binding site and improving the long-term robustness of molecular POCT as SARS-CoV-2 continues to evolve [10,41,42,43].

Compared with antigen-based assays, molecular POCT platforms generally exhibit greater resilience to viral evolution because they target conserved genomic regions and employ multiplex detection strategies. CDC genomic surveillance from May 2023 through September 2024 documented the progressive replacement of XBB lineages by JN.1 and its descendants, illustrating the continuous evolutionary pressure imposed on diagnostic targets. Supporting the effectiveness of conserved-target approaches, Almeida et al. demonstrated that RT-LAMP assays targeting the N and E genes retained robust analytical sensitivity for Omicron detection despite extensive genomic mutations, achieving a limit of detection of approximately 0.4 copies/µL. Similarly, the CRISPR-based OmiCrisp assay successfully detected successive Omicron sub-lineages, including BA.5, BA.2.75, XBB.1, XBB.1.5, and JN.1, through conserved ORF1ab and N gene targets. Collectively, these findings support the continued use of molecular POCT platforms as among the most mutation-resilient diagnostic approaches for ongoing SARS-CoV-2 surveillance and variant detection [10,43,44,45].

**Table 1:** Different commercial PCR-based rapid POCT. Note: Performance metrics should be interpreted in context, as they vary depending on study design, sample size, reference standards, and patient population; therefore, comparisons are indicative rather than absolute.

Test	Mechanism	Time (min)	Result Reading	Sample	Approval	+/− Agreement (Percentage)	LOD	Availability	Clinical utility	References
IDNOW COVID-19	Nicking endonuclease amplification reaction	5–13	Device	NS/OS/NPS	FDA(EUA)	93.3 98.4	125 genomes equivalents/mL	Clinical	Emergency departments, urgent care	[46,47,48,49]
VitaPCR™ Platform	Real-time PCR	20 min	Device	NPS/OS	CE	100 100	2730 copies/mL	Clinical	Near-patient hospital testing	[50,51]

PCR = polymerase chain reaction, NS = nasal swab, OS = oropharyngeal swab, NPS = nasopharyngeal swab, FDA = food and drug administration, EUA = emergency use authorization, CE = Conformite Europeenne. Table values are presented as reported in the original studies. Direct comparison across platforms should be interpreted cautiously because studies differed substantially in sample size, patient characteristics, specimen type, symptom status, timing of sample collection, viral load distribution, circulating variants, clinical setting, reference standards, and study design. Therefore, reported sensitivity and specificity values should be considered indicative rather than directly comparable measures of performance.

### 3.2. Ag-Based POCT

**Figure 2:**
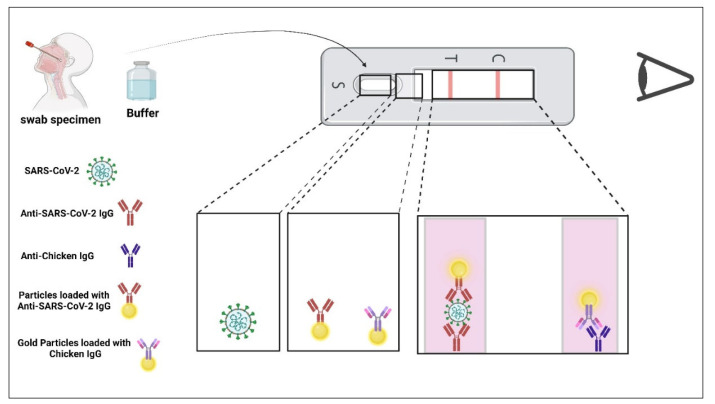
Schematic representation of a lateral flow immunoassay for SARS-CoV-2 antigen detection. A swab specimen mixed with buffer is applied to the sample (S) pad. If SARS-CoV-2 antigens are present, they bind to gold nanoparticles coated with anti-SARS-CoV-2 IgG, forming antigen–antibody complexes that migrate along the strip. These complexes are captured at the test (T) line by immobilized anti-SARS-CoV-2 IgG, producing a visible signal. The control (C) line contains anti-chicken IgG that captures chicken IgG-conjugated gold particles, ensuring proper flow and assay functionality. Created in BioRender. Hetta, H. (2026) https://BioRender.com/bu64h2i (accessed on 10 June 2026).

Antigen-based point-of-care tests detect specific viral proteins, most commonly the nucleocapsid protein of SARS-CoV-2, using immunoassay-based techniques. These assays are typically performed using lateral flow formats (Figure 2) and provide results within 10–20 min, making them highly suitable for rapid screening and large-scale testing [17,21,32,52,53,54,55].

Compared to molecular methods, antigen-based POCT is simpler to perform, does not require specialized instrumentation, and is generally more cost-effective. Commercial antigen tests (Table 2) are widely used in decentralized settings, including community screening, emergency departments, and at-home testing [56,57].

However, antigen assays generally exhibit lower sensitivity than molecular diagnostics, particularly in asymptomatic individuals or during early and late stages of infection when viral loads are low. Their performance is strongly dependent on viral load, with higher sensitivity observed during the acute phase of infection when viral shedding is at its peak. Variability in sample collection and operator technique can further influence test accuracy in real-world settings [56,58].

Despite these limitations, antigen-based POCT plays a critical role in public health strategies due to its rapid turnaround time and scalability. In high-prevalence settings or symptomatic individuals, these tests can provide timely identification of infectious cases. However, negative results, particularly in clinically suspected cases, may require confirmation using molecular methods [21].

Overall, antigen-based POCT represents a trade-off between diagnostic sensitivity and operational efficiency. While less sensitive than molecular assays, particularly in low viral load infections, their rapid turnaround time, low cost, and ease of deployment make them well suited for large-scale screening and outbreak control. Consequently, antigen tests are best positioned as frontline screening tools in high-throughput or resource-limited settings, whereas molecular assays remain the reference standard for confirmatory diagnosis [17,58].

A major concern for antigen-based POCT is whether rapid antigen tests (RATs) retain adequate diagnostic performance against successive Omicron sub-lineages. Current evidence is generally reassuring. A large retrospective study involving 8620 clinical encounters in Singapore demonstrated that RATs maintained an overall sensitivity of 84.6% (95% CI, 83.3–85.7%) and specificity of 99.4% (95% CI, 99.1–99.6%) during periods dominated by the BA.2, BA.5, and XBB.1 variants, with no significant differences in diagnostic performance between sub-lineages. Similarly, an analytical evaluation of seven Japanese regulatory-approved RATs showed comparable sensitivity across BA.5, BA.2.75, BF.7, XBB.1, and BQ.1.1 variants, suggesting that mutations accumulated during Omicron evolution have not substantially compromised antigen recognition. A recent systematic review and meta-analysis including 50,897 individuals further reported pooled sensitivity and specificity estimates of 0.77 and 1.00, respectively, while confirming that diagnostic sensitivity remains highly dependent on viral load, declining markedly in specimens with higher cycle threshold values. Likewise, CDC surveillance during continued Omicron circulation demonstrated that although rapid antigen tests remain effective in identifying potentially transmissible infections, their sensitivity is substantially lower than RT-PCR, particularly in low viral load infections. Collectively, these findings indicate that currently available RATs continue to provide reliable detection of circulating Omicron sub-lineages in patients with moderate-to-high viral loads, although reduced sensitivity during early infection and in asymptomatic individuals remains an inherent limitation independent of variant evolution [21,59,60,61,62,63].

Beyond clinical performance evaluation, recent developments have focused on improving the analytical and operational characteristics of antigen-based testing platforms. Recent developments have led to digital lateral flow assays (digital LFAs), which combine conventional immunochromatographic test strips with portable electronic readers. Unlike traditional visual interpretation, digital readers quantify signal intensity, reducing user-dependent variability and enabling semi-quantitative analysis. These systems represent an intermediate diagnostic platform between conventional antigen tests and biosensor-based technologies, offering improved analytical consistency while preserving operational simplicity. However, further large-scale clinical validation is required before widespread implementation in routine diagnostic pathways [64,65,66].

**Table 2:** Different commercial antigen-based rapid POCT. Note: Performance metrics should be interpreted in context, as they vary depending on study design, sample size, reference standards, and patient population; therefore, comparisons are indicative rather than absolute.

Test	Mechanism	Time (min)	Result Reading	Sample	Approved by	+/− Agreement (Percentage)	Availability	References
Abbott BinaxNOW COVID-19 Ag Card Home Test	LFIA	15	Naked eye	NS	FDA (EUA)	91.7 100	Active	[67,68,69,70]
Quick Vue At-Home OTC COVID-19 Test	LFIA	10	Naked eye	NS	FDA (EUA)	83.5 99.2	Active	[68,71]
Celltrion DiaTrust COVID-19 Antigen Rapid Test	LFIA	15	Naked eye	NPS	FDA (Former EUA)	93.33 99.03	Inactive	[72]
BD Veritor System	LFIA	15	Device	NS	FDA (Former EUA)	83.4 99.7	Inactive	[73,74,75,76]
Lumira Dx SARS-CoV-2 Ag Test	MIFIA	12	Device	NS	FDA(EUA), CE	97.6 96.6	Clinical	[77,78]
Sofia SARS Antigen FIA	LF-IFIA	15	Device	NPS/NS	FDA(EUA), CE	96.7 100	Clinical	[79,80]

LFIA = lateral flow immunoassay, MIFIA = microfluidic immunofluorescence immunoassay, LF-IFIA = lateral flow immunofluorescence-based immunoassay, NS = nasal swab, NPS = nasopharyngeal swab, FDA = food and drug administration, EUA = emergency use authorization, CE = Conformite Europeenne. Table values are presented as reported in the original studies. Direct comparison across platforms should be interpreted cautiously because studies differed substantially in sample size, patient characteristics, specimen type, symptom status, timing of sample collection, viral load distribution, circulating variants, clinical setting, reference standards, and study design. Therefore, reported sensitivity and specificity values should be considered indicative rather than directly comparable measures of performance.

### 3.3. Ab-Based POCT

**Figure 3:**
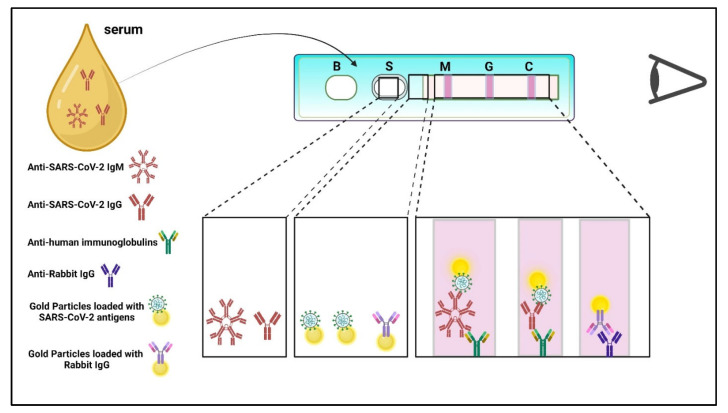
Schematic representation of a lateral flow immunoassay (LFIA) for the detection of SARS-CoV-2-specific antibodies (IgM and IgG) in serum. The test strip contains separate detection lines for IgM (M), IgG (G), and a control (C). Anti-SARS-CoV-2 antibodies in the serum bind to gold particles conjugated with viral antigens and are captured by anti-human immunoglobulins at the respective test lines. The control line captures gold particles conjugated with rabbit IgG to verify test validity. Created in BioRender. Hetta, H. (2026) https://BioRender.com/bu64h2i (accessed on 10 June 2026).

Antibody-based point-of-care tests (POCTs) differ fundamentally from antigen and molecular diagnostics because they rely on detection of the host immune response rather than direct identification of the virus. Following SARS-CoV-2 infection, a serological window period occurs before immunoglobulins become detectable, creating a risk of false-negative results during early infection. IgM antibodies generally appear first, followed by IgG antibodies after approximately 1–3 weeks, with IgG persisting for a longer duration. These assays are most commonly implemented using the lateral flow immunoassay (LFIA) format, enabling rapid detection of IgM/IgG in whole blood, serum, or plasma within 10–20 min. Consequently, antibody-based POCTs are more suitable for retrospective assessment, seroprevalence studies, and epidemiological surveillance rather than early clinical diagnosis (Figure 3; Table 3) [81,82,83,84,85,86,87,88,89,90].

Unlike molecular and antigen-based diagnostics, which identify active viral infection, antibody tests reflect prior exposure to the virus. Because immunoglobulins develop only after immune activation, antibody-based POCT is not suitable for early detection of acute infection, particularly during the first days following viral exposure [85,91].

Commercial antibody-based POCT platforms have been widely used for seroprevalence studies, epidemiological surveillance, and assessment of immune response following infection or vaccination. However, their diagnostic performance varies depending on the timing of testing relative to infection and individual immune variability [88,92].

A key limitation of antibody-based assays is their reduced clinical utility in acute diagnosis due to delayed seroconversion. In addition, cross-reactivity with other human coronaviruses and variability in assay specificity may lead to false-positive results. These factors limit their reliability as standalone diagnostic tools for confirming active SARS-CoV-2 infection [91,93].

Overall, antibody-based POCT serves a complementary role in SARS-CoV-2 diagnostics rather than a primary diagnostic function. In contrast to molecular and antigen-based tests designed to detect current infection, antibody assays are more appropriate for retrospective analysis and population-level studies. Their value lies in understanding exposure patterns and immune responses, but their limitations in early detection and diagnostic specificity restrict their use in clinical decision-making for acute cases [88,92].

**Table 3:** Different commercial antibody-based rapid POCT. Note: Performance metrics should be interpreted in context, as they vary depending on study design, sample size, reference standards, and patient population; therefore, comparisons are indicative rather than absolute.

Test	Target	Mechanism	Time (min)	Result Reading	Sample	Approved by	+/− Agreement (Percentage)	Availability	References
ACON SARS-CoV-2 IgG/IgM Rapid Test	IgG & IgM	LFIA	15	Naked eye	whole blood, plasma, and serum	FDA (EUA)	99.1 98.2	Active	[94,95,96]
WANTAI SARS-CoV-2 Ab Rapid Test	IgG, IgM, IgA	LF-CGIA	15	Naked eye	Serum/plasma (Dipotassium EDTA, lithium heparin and sodium citrate)/Venous whole blood	FDA (EUA), CE, TGA	94.70 98.89	Active	[97]
BioCheck SARS-CoV-2 IgG and IgM Combo test	IgM and IgG	CLIA	30	Machine	Serum	FDA (EUA), CE	99.1 97.2	Clinical	[98]
SARS-CoV-2 Antibody Test	IgM and IgG	LF-CGIA	15	Naked eye	Whole blood/serum/plasma	FDA (EUA), CE, TGA, NMPA	45.2 81.8	Active	[99,100,101,102]
Diagnostic Kit for IgM/IgG Antibody to Coronavirus (SARS-CoV-2)	IgM and IgG	LF-CGIA	15	Naked eye	Serum/plasma/Venous whole blood	CE, NMPA	90.6 99.2	Active	[103]

LFIA = lateral flow immunoassay, LF-CGIA = lateral flow colloidal gold immunoassay, CLIA = Chemiluminescence Immunoassay, TRF-LFIA = time-resolved fluorescence lateral flow immunochromatographic assay FDA = food and drug administration, EUA = emergency use authorization, CE = Conformite Europeenne, NMPA = National Medical Products Administration, TGA = Therapeutic Goods Administration. Table values are presented as reported in the original studies. Direct comparison across platforms should be interpreted cautiously because studies differed substantially in sample size, patient characteristics, specimen type, symptom status, timing of sample collection, viral load distribution, circulating variants, clinical setting, reference standards, and study design. Therefore, reported sensitivity and specificity values should be considered indicative rather than directly comparable measures of performance.

### 3.4. Biosensor-Based POCT

Biosensor-based point-of-care testing has emerged as a distinct diagnostic category that leverages nanomaterial-enhanced electrochemical, optical, and electronic transduction mechanisms for rapid SARS-CoV-2 detection. Unlike conventional molecular or antigen assays, these platforms enable direct physicochemical signal conversion with minimal sample preparation. Although most systems remain at the prototype stage, rapid advances in plasmonic, electrochemical, and graphene-based sensing have driven a clear technological progression toward practical POCT implementation [52,104,105,106,107,108].

The rapid evolution of nanomaterial-enabled biosensing has significantly reshaped point-of-care testing strategies for SARS-CoV-2. Early efforts were largely driven by plasmonic nanostructures exploiting localized surface plasmon resonance (LSPR), where light–matter interactions at metallic nanoparticle surfaces enabled analytically sensitive, label-free detection. A notable example is the colorimetric assay developed by Moitra et al., in which thiol-modified antisense oligonucleotides were conjugated to gold nanoparticles to target the viral N gene. Upon hybridization and subsequent ribonuclease H activity, nanoparticle aggregation produced a visible color change within 10 min, demonstrating how plasmonic effects could be translated into naked-eye diagnostics without instrumentation [109,110,111,112,113].

Building on this optical foundation, researchers sought to enhance hybridization efficiency and analytical sensitivity by coupling plasmonic sensing with photothermal effects. Qiu et al. demonstrated that thermoplasmonic heating generated by two-dimensional gold nanoislands could locally elevate temperature to accelerate nucleic acid hybridization while simultaneously enabling LSPR sensing, achieving picomolar-level detection limits. In parallel, Murugan et al. translated plasmonic sensing into a fiber-optic absorbance platform capable of wash-free detection of viral N protein directly from saliva, showing how plasmonics could move from proof-of-concept optics toward practical clinical sampling [114,115].

As optical plasmonic systems matured, integration with microfluidics became the next logical step to improve sample handling and assay automation. Funari et al. introduced an opto-microfluidic chip containing gold nanospikes, where immunoglobulin binding produced measurable resonance wavelength shifts. This design illustrated how plasmonic sensing could be embedded within compact fluidic architectures, enabling quantitative results in under 30 min with minimal user intervention [116].

Despite the high sensitivity of optical systems, their dependence on optical components and alignment motivated a transition toward electrochemical transduction, which offers simpler instrumentation, lower cost, and easier miniaturization. Advances in nanomaterials such as graphene, gold nanoparticles, and carbon nanostructures enabled this shift. Md. Ali et al. employed 3D-printed reduced graphene oxide electrodes integrated into microfluidics for femtomolar immunoglobulin detection, while Fabiani et al. combined magnetic beads with carbon black electrodes to eliminate washing steps through magnetic pre-concentration, enhancing robustness against interference [117,118].

To further reduce cost and complexity, paper-based electrochemical platforms emerged. Alafeef et al. immobilized probes onto gold-nanoparticle-modified paper electrodes to detect viral nucleic acids within 5 min across a wide dynamic range [119]. Coupling such sensors with smartphone-compatible readers, as demonstrated by Zhao et al., addressed the critical requirement of data transmission and real-time reporting for at-home diagnostics [120].

Electrochemical miniaturization continued with portable graphene and gold-nanoparticle electrodes integrated with wireless modules. Platforms such as the RapidPlex system demonstrated multiplexed detection of viral markers and immune responses. Expanding the concept further, Miripour et al. showed that electrochemical sensing could even monitor reactive oxygen species in sputum, leveraging infection-induced oxidative stress as an indirect diagnostic marker measurable in under 30 s [121,122,123].

Beyond liquid samples, researchers explored non-invasive breath analysis. Shan et al. functionalized gold nanoparticles with organic ligands to detect volatile organic compounds in exhaled air through resistance changes. Although promising for rapid screening, environmental and physiological confounders remain challenges for real-world deployment [124].

**Figure 4:**
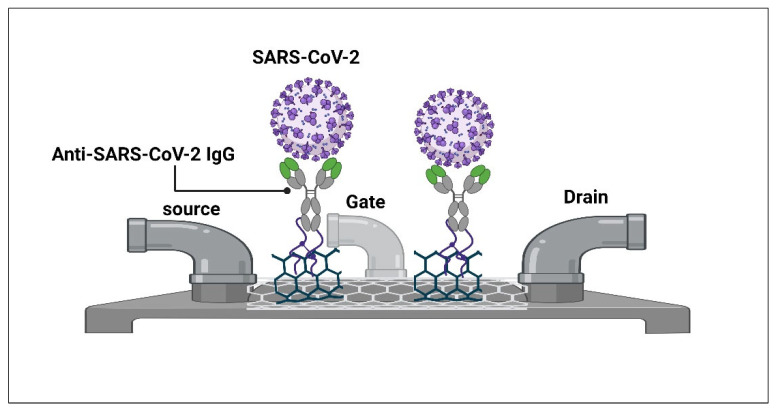
Schematic representation of a field-effect transistor (FET)-based biosensor for SARS-CoV-2 detection. Anti-SARS-Cov-2 IgG antibodies are immobilized on the graphene surface between the source and drain electrodes. Binding of the viral particles to the antibodies alters the electrical signal at the gate region, enabling label-free and real-time detection of the virus. Created in BioRender. Hetta, H. (2026) https://BioRender.com/bu64h2i (accessed on 10 June 2026).

At the most advanced end of label-free electronic sensing, field-effect transistor (FET) biosensors (Figure 4) represent a culmination of nanomaterial integration. Seo et al. functionalized graphene sheets with antibodies against the viral spike protein, achieving femtogram-level detection directly from nasopharyngeal swabs without labeling or complex preparation. This approach exemplifies how nanomaterials enable direct electrical readout of biomolecular interactions, offering a robust foundation for rapid, precise point-of-care diagnosis [125].

Together, these developments illustrate a clear technological progression: from plasmonic optical detection, to optothermal enhancement, to microfluidic integration, to electrochemical miniaturization, to non-invasive sensing, and finally to fully electronic graphene-based biosensors—each step addressing limitations of the previous generation while moving closer to practical, rapid, and accessible point-of-care diagnostics.

Although biosensor-based diagnostics demonstrate impressive analytical sensitivity under laboratory conditions, many platforms remain at early stages of technological development. Importantly, analytical performance metrics obtained using synthetic samples, purified nucleic acids, or controlled laboratory conditions should not be interpreted as equivalent to clinical diagnostic accuracy. Translation into real-world clinical settings requires additional validation across diverse patient populations, specimen types, and operational environments.

Another major challenge involves long-term stability and shelf life. Many biosensors listed in Table 4 rely on biological recognition elements such as antibodies, aptamers, or enzymes that may degrade during storage or transportation. Consequently, maintaining analytical performance outside controlled laboratory conditions remains a significant barrier to large-scale deployment. Standardization, manufacturability, regulatory approval, and cold-chain requirements represent critical considerations for future clinical implementation [126,127].

## 4. Artificial Intelligence (AI)-Assisted Diagnosis for COVID-19

The integration of AI and POCT diagnostics aims to improve the speed and accuracy of disease diagnosis. AI can analyze data from various devices and enable personalized care. It can be embedded in PoC testing devices for advanced analyses, such as image processing. Local device networks and decentralized AI approaches extend POC diagnostics. This integration has the potential to reduce disparities, enhance care quality, and save lives. Research articles have explored AI’s application in POC diagnostics, including accurate diagnosis of pediatric pneumonia, autonomous AI for detecting diabetic eye disease, improved lesion detection in medical images, automated segmentation of dermatoscopy images for skin lesion diagnosis, and the detection and analysis of circulating tumor cells in cancer diagnosis. These studies demonstrate AI’s potential to enhance diagnostics and improve patient outcomes in POC settings [129].

Artificial intelligence (AI) has emerged as a complementary technology capable of enhancing diagnostic accuracy, automation, and clinical decision-making across multiple healthcare settings [130,131]. However, not all AI applications discussed in the context of COVID-19 represent true point-of-care testing modalities. Portable and decentralized systems such as AI-assisted point-of-care ultrasound (AI-POCUS), smartphone-based diagnostic applications, and AI-integrated POCT interpretation platforms are more closely aligned with the POCT concept than centralized imaging modalities such as computed tomography (CT) and conventional radiography.

Accordingly, AI technologies discussed in this review should be viewed as complementary diagnostic support tools rather than direct replacements for established SARS-CoV-2 testing methods.

New research presented at the European Respiratory Society International Congress suggests that AI can accurately detect COVID-19 infection by analyzing people’s voices through a mobile phone application (app.). In a preliminary study, a voice-based model showed higher reported accuracy than lateral flow tests in that dataset; however, external validation and prospective clinical evaluation remain limited. By utilizing data from a crowdsourced COVID-19 app, the Sounds app was developed by the University of Cambridge. The researchers collected audio samples from both healthy and infected participants. They employed Mel-spectrogram analysis, a voice analysis technique, to identify specific voice features associated with COVID-19. Among the different AI models built, the Long-Short Term Memory (LSTM) model performed the best, achieving an overall accuracy of 89%, sensitivity of 89%, and specificity of 83%. These results indicate a significant improvement over the lateral flow test. However, the findings still require validation with a larger sample size. The researchers are also further analyzing the voice parameters that influence the AI model’s accuracy [132].

Among AI applications that are more closely aligned with the point-of-care testing paradigm, portable ultrasound systems integrated with artificial intelligence have demonstrated considerable potential for rapid bedside assessment of COVID-19-related pulmonary abnormalities. A study evaluated the effectiveness of a pocket-sized ultrasound device, used by a novice observer, in detecting pneumonia compared to CT scans. The 12-zone AI-POCUS showed a high accuracy of 94.5% in detecting pneumonia at the patient level. The simplified 8-zone scan had a slightly lower accuracy of 83.9%. Despite being performed by a novice, the AI-POCUS showed excellent agreement with CT-validated pneumonia. Overall, the study demonstrated the reliability of AI-POCUS in detecting pneumonia, even in inexperienced hands [133]. Nevertheless, most AI studies remain exploratory and should not yet be considered equivalent to validated diagnostic assays.

In addition to AI, Modern POCT platforms increasingly incorporate wireless communication capabilities and integration with Internet of Medical Things (IoMT) infrastructures. These technologies enable automatic transmission of diagnostic results to healthcare providers, public health surveillance systems, and electronic medical records. Such connectivity facilitates real-time epidemiological monitoring and rapid outbreak response [134,135].

However, increased connectivity also raises concerns regarding patient privacy, cybersecurity, data ownership, and regulatory compliance. Future POCT development must therefore balance diagnostic innovation with robust safeguards for data security, confidentiality, and ethical use of healthcare information [136].

## 5. Regulatory Considerations and Quality Control

The rapid deployment of SARS-CoV-2 diagnostic tests was facilitated by emergency regulatory pathways such as Emergency Use Authorization (EUA) and CE marking. While these mechanisms enabled timely access to testing, they also contributed to variability in validation standards and reported performance [17].

In addition, differences in study design, evaluation protocols, and reporting criteria have led to challenges in comparing diagnostic performance across platforms. Post-market surveillance and continuous performance monitoring are therefore essential to ensure reliability over time. The development of standardized validation frameworks and harmonized regulatory guidelines remains critical for improving the consistency and quality of POCT technologies [21].

## 6. Implementation and Challenges

Despite the advantages of POCT, several challenges limit its widespread implementation. Cost remains a significant barrier, particularly for molecular-based platforms requiring specialized devices and consumables. Infrastructure limitations, including supply chain constraints and device availability, further restrict deployment in resource-limited settings. In addition, proper training of healthcare personnel and end-users is essential to ensure accurate test performance and interpretation. Variability in user handling and adherence to testing protocols can significantly affect diagnostic accuracy in real-world settings. Addressing these practical challenges is essential to maximize the effectiveness and scalability of POCT technologies [17,137].

## 7. Comparative Analysis of POCT Modalities for SARS-CoV-2

The various categories of point-of-care testing (POCT) for SARS-CoV-2 differ substantially in their diagnostic performance, operational requirements, and clinical applicability. Molecular-based assays remain the most analytically sensitive, particularly in early infection, but are generally associated with higher cost, device dependency, and lower scalability compared to other POCT formats [17]. Because the reviewed studies differed substantially in specimen type, reference standard, patient population, viral-load distribution, circulating variants, and study design, comparisons across modalities should be interpreted as qualitative assessments of technology characteristics and maturity rather than direct head-to-head performance comparisons.

In contrast, antigen-based tests offer rapid results and ease of use, making them suitable for large-scale screening; however, their sensitivity is strongly dependent on viral load and is reduced in asymptomatic individuals or early-stage infection. Antibody-based assays, while useful for seroprevalence and retrospective analysis, have limited utility in acute diagnosis due to delayed seroconversion and variability in immune response [21,138].

Emerging biosensor-based and AI-assisted platforms demonstrate promising analytical performance and rapid detection capabilities; however, their clinical utility remains constrained by limited large-scale validation, lack of standardization, and lower technology readiness levels compared with molecular and antigen-based POCT. While molecular and antigen assays have undergone extensive clinical validation and regulatory review, many biosensor and AI-enabled systems remain supported primarily by proof-of-concept studies, pilot investigations, or limited clinical validation cohorts. Consequently, comparisons between clinically implemented technologies and emerging experimental platforms should be interpreted cautiously [139,140,141].

Across all modalities, diagnostic performance in real-world settings is influenced by factors such as sample quality, operator variability, and disease prevalence. In addition, the emergence of SARS-CoV-2 variants may differentially impact test performance, particularly for assays targeting variable viral proteins [17].

From a regulatory and implementation perspective, molecular and antigen-based POCT have achieved broader clinical adoption due to established validation frameworks and regulatory approvals, whereas antibody and biosensor-based platforms face greater challenges related to standardization and real-world deployment. Cost, infrastructure requirements, and user training further influence the feasibility of widespread implementation, particularly in resource-limited settings [17,138].

**Figure 5:**
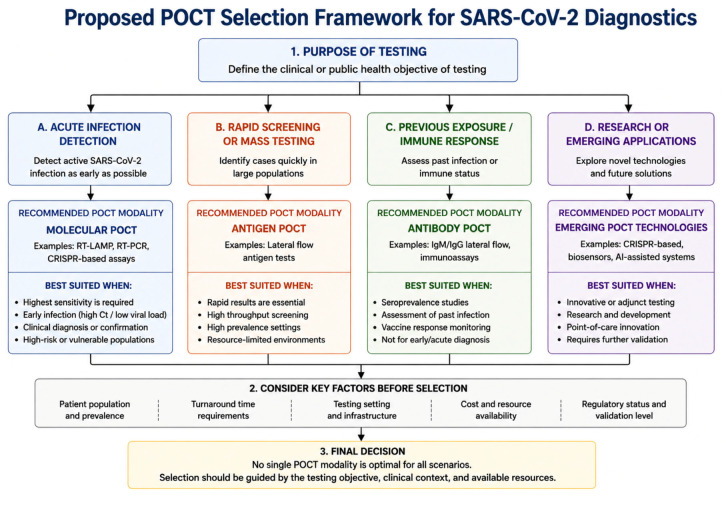
Proposed framework for selecting SARS-CoV-2 point-of-care testing (POCT) modalities according to diagnostic objectives, clinical context, resource availability, and technology readiness. Molecular POCT is most suitable when maximum diagnostic sensitivity is required, whereas antigen-based POCT supports rapid large-scale screening. Antibody-based testing is primarily intended for serosurveillance and assessment of prior exposure. Emerging technologies, including CRISPR-based diagnostics, biosensors, and AI-assisted systems, represent promising future approaches but currently require additional clinical validation before widespread implementation. The proposed framework is intended as a conceptual guide based on current evidence and should not be interpreted as a substitute for assay-specific performance evaluation, regulatory guidance, or clinical judgment. Created in BioRender. Hetta, H. (2026) https://BioRender.com/bu64h2i (accessed on 10 June 2026).

Overall, no single POCT modality is optimal across all clinical scenarios (Figure 5). Instead, the selection of an appropriate diagnostic approach depends on the intended use, testing context, and balance between sensitivity, speed, and accessibility. Due to heterogeneity across studies and diagnostic platforms, a qualitative synthesis approach was used to compare key characteristics of different POCT modalities (Table 5).

## 8. Conclusions

Point-of-care testing (POCT) has become an essential component of SARS-CoV-2 diagnostic strategies, enabling rapid, decentralized, and accessible testing across diverse healthcare settings. The unprecedented diagnostic demands of the COVID-19 pandemic accelerated the development and deployment of a wide range of POCT technologies, resulting in significant advances in molecular diagnostics, antigen detection, serological testing, biosensor platforms, and artificial intelligence (AI)-assisted approaches.

The evidence reviewed indicates that molecular POCT platforms currently provide the most robust combination of analytical sensitivity, specificity, and resilience to viral genetic variation, making them the preferred option for accurate detection of active infection. Antigen-based assays remain valuable for rapid screening, large-scale testing programs, and resource-limited settings because of their affordability, simplicity, and rapid turnaround time, despite reduced sensitivity in certain clinical scenarios. Antibody-based tests continue to support serosurveillance, epidemiological investigations, and assessment of previous exposure but are not suitable as standalone tools for acute infection diagnosis.

Emerging technologies, including CRISPR-based diagnostics, advanced biosensors, nanotechnology-enabled platforms, and AI-assisted systems, demonstrate considerable potential to expand the capabilities of decentralized diagnostics. However, many of these technologies remain at the proof-of-concept or early clinical validation stage, and their reported performance should be interpreted within the context of limited real-world evaluation, heterogeneous study designs, and evolving regulatory frameworks. Consequently, additional clinical validation, standardization, and implementation studies are required before widespread adoption can be recommended.

Importantly, no single POCT modality is universally optimal for all clinical and public health applications. Rather than representing competing technologies, molecular, antigen, antibody, biosensor, and AI-assisted approaches should be viewed as complementary components of an integrated diagnostic ecosystem. The selection of an appropriate testing strategy should be guided by clinical objectives, disease prevalence, resource availability, turnaround-time requirements, and the balance between diagnostic performance and accessibility. Collectively, these technologies have transformed decentralized infectious disease diagnostics and established a foundation for more responsive and resilient healthcare systems.

## 9. Future Perspectives

One of the most important trends shaping the future of respiratory point-of-care testing (POCT) is the transition from single-pathogen assays toward multiplex respiratory panels capable of simultaneously detecting multiple viral pathogens from a single patient specimen. Because SARS-CoV-2, influenza A, influenza B, and respiratory syncytial virus (RSV) frequently present with overlapping clinical manifestations, distinguishing among these infections based on symptoms alone remains challenging. Multiplex POCT platforms address this limitation by enabling rapid syndromic diagnosis, thereby supporting timely clinical decision-making, targeted antiviral therapy, infection-control measures, and improved antimicrobial stewardship [11,12].

The COVID-19 pandemic accelerated the development and clinical adoption of multiplex molecular assays designed to detect SARS-CoV-2 alongside a broad spectrum of respiratory pathogens, including influenza viruses, respiratory syncytial virus (RSV), adenoviruses, parainfluenza viruses, rhinoviruses/enteroviruses, and seasonal coronaviruses, as well as underrecognized respiratory viruses such as human metapneumovirus (HMPV). Several commercially available multiplex molecular platforms, including the BioFire respiratory panel, Xpert Xpress CoV-2/Flu/RSV Plus, and QIAstat-Dx respiratory panels, have demonstrated the feasibility of providing highly accurate differential diagnosis within a single integrated workflow. Beyond improving patient management, these multiplex approaches facilitate surveillance of co-circulating respiratory pathogens and support public health responses during seasonal epidemics and emerging outbreaks [147,148,149,150,151,152,153,154,155,156,157,158,159,160].

Future multiplex POCT systems are expected to expand pathogen coverage while maintaining rapid turnaround times, high analytical sensitivity, and operational simplicity. Advances in microfluidics, biosensor engineering, nanotechnology, isothermal amplification, and CRISPR-based detection systems are enabling the development of portable syndromic testing platforms with increasing multiplex capacity and reduced infrastructure requirements. At the same time, continued optimization of primer and probe design, target selection, and assay architecture will remain essential for maintaining diagnostic accuracy in the face of ongoing viral evolution and the emergence of novel variants. Technologies capable of rapid adaptation to genetic changes are likely to play an increasingly important role in future outbreak preparedness and response.

The integration of multiplex POCT with digital health infrastructure represents another important direction for future development. Connectivity-enabled diagnostic devices, Internet of Medical Things (IoMT) platforms, cloud-based reporting systems, and artificial intelligence (AI)-assisted decision-support tools have the potential to facilitate real-time disease monitoring, automated data analysis, and more effective public health interventions. Integration of multiplex respiratory panels with digital surveillance networks may further enhance outbreak detection, epidemiological monitoring, and pandemic preparedness. However, successful implementation will require careful attention to data security, patient privacy, interoperability, and regulatory compliance.

Despite substantial technological progress, important challenges remain. Multiplex assays must balance broad pathogen coverage with maintenance of analytical sensitivity and specificity across multiple targets, while minimizing target competition and assay complexity. In addition, many emerging diagnostic platforms continue to face barriers related to large-scale manufacturing, reproducibility, quality assurance, regulatory approval, cost-effectiveness, and clinical validation in diverse populations and real-world settings. Addressing these challenges will require multidisciplinary collaboration among researchers, clinicians, industry partners, regulatory agencies, and public health organizations.

Ultimately, the future of POCT lies not in a single transformative technology but in the integration of complementary diagnostic approaches that combine multiplex detection, high analytical performance, operational simplicity, digital connectivity, and broad accessibility. Continued innovation, supported by rigorous clinical evaluation and evidence-based implementation, will be critical for strengthening global preparedness against future infectious disease outbreaks and enhancing the resilience of healthcare systems worldwide.

## Figures and Tables

**Table 4:** Representative Biosensor-Based POCT Technologies for SARS-CoV-2 Detection. Note: Performance metrics should be interpreted in context, as they vary depending on study design, sample size, reference standards, and patient population; therefore, comparisons are indicative rather than absolute.

Test	Target	Material Used	Time	Sample	Limit of Detection	Availability	References
Surface plasmon resonance and colorimetric assay	Nucleic acid	Gold nanoparticles	10 min	Isolated RNA	180 ng/mL	Research	[109]
Plasmonics and photothermal effect	Nucleic acid	Gold nanoislands	Real-time detection	Synthetic RNA targets and extracted clinical RNA samples	0.22 pM	Research	[114]
Opto-microfluidic Chip	Antibodies	Gold nanospikes	30 min	Diluted human plasma	0.5 pM	Experimental	[116]
3D electrochemical Sensor	Antibodies	Reduced-graphene-oxide nanoflakes	Seconds	Recombinant SARS-CoV-2 antibodies in PBS (spiked laboratory samples)	2.8 fM	Experimental	[117]
Magnetic beads based biosensor	Spike (S) protein and nucleocapsid (N) protein	Magnetic beads and carbon black-based electrodes	30 min	Untreated saliva	19 ng/mL (S protein), 8 ng/mL (N protein)	Experimental	[118]
Paper-based electrochemical sensor	Antibodies	Graphene-based materials	30 min	Human serum	IgG: 0.96 ng/mL; IgM: 0.14 ng/mL	Experimental	[128]
Paper-based electrochemical sensor	Nucleic acid	Gold nanoparticles	<5 min	synthetic + clinical samples	6900 copies/mL	Experimental	[119]
Electrochemical Sensor	Reactive oxygen species	Multi-wall carbon nanotubes	<30 s	Fresh sputum	NR	Experimental	[123]
Nanomaterials-based breath sensor	Disease-specific biomarkers	Gold nanoparticles	Seconds	Exhaled breath	NR	Experimental	[124]
Field-effect transistor	Spike (S) Protein	Graphene sheet	Real-time detection	Clinical samples from COVID-19 patients	1 fg/mL	Experimental	[125]

LOD = limit of detection; NR = not reported; pM = picomolar (10^−12^ M); fM = femtomolar (10^−15^ M); ng/mL = nanogram per milliliter; fg/mL = femtogram per milliliter; copies/mL = viral genome copies per milliliter; SPR = surface plasmon resonance; RNA = ribonucleic acid; IgG = immunoglobulin G; IgM = immunoglobulin M; ROS = reactive oxygen species; S protein = spike protein; N protein = nucleocapsid protein. LOD values are reported as originally described by each study and are therefore not directly comparable. Table values are presented as reported in the original studies. Direct comparison across platforms should be interpreted cautiously because studies differed substantially in sample size, patient characteristics, specimen type, symptom status, timing of sample collection, viral load distribution, circulating variants, clinical setting, reference standards, and study design. Therefore, reported sensitivity and specificity values should be considered indicative rather than directly comparable measures of performance.

**Table 5:** Comparative overview of SARS-CoV-2 POCT modalities (qualitative synthesis). Note: Performance metrics should be interpreted in context, as they vary depending on study design, sample size, reference standards, and patient population; therefore, comparisons are indicative rather than absolute.

Feature	Molecular POCT	Antigen POCT	Antibody POCT	Biosensor-Based POCT	References
Target	Viral RNA	Viral proteins	Host antibodies (IgM/IgG)	Viral RNA, proteins, or host biomarkers	[142,143]
Primary clinical use	Detection of active infection (including early stage)	Rapid screening of active infection	Assessment of past exposure or immune response	Emerging applications (detection of viral or host markers)	[89,142,144]
Analytical sensitivity	Generally high	Moderate and dependent on viral load	Low in early infection; increases over time	High in analytical settings; limited clinical validation	[89,142,144]
Specificity	Generally high	Generally high but variable across assays	Variable; may be affected by cross-reactivity	High in experimental settings	[89,145]
Time to result	~15–30 min	~10–20 min	~10–20 min	Seconds to minutes (depending on platform)	[142,143]
Impact of variants	Potentially limited due to conserved targets, but assay-dependent	More susceptible to performance variation due to protein mutations	May be influenced by antigenic variation affecting antibody binding	Not well established; depends on target selection	[146]
Performance in real-world settings	Generally high but influenced by sample quality and testing conditions	Reduced sensitivity compared to molecular methods, especially in low viral load cases	Highly variable depending on timing of testing and population	Not well established due to limited large-scale clinical studies	[89,142,144]
Regulatory status	Widely authorized for clinical use	Widely authorized for clinical use	Widely available; variable validation quality	Limited regulatory approval; mostly experimental	[142,143,144]
Cost considerations	Relatively high	Generally low	Generally low	Variable and not well standardized	[143,144]
Infrastructure requirements	Requires dedicated devices or platforms	Minimal; suitable for decentralized settings	Minimal	Variable; often requires specialized components	[143,144]
Training requirements	Moderate	Low	Low	Moderate to high (depending on system complexity)	[143,144]
Scalability	Moderate	High	High	Currently limited	[142,144]
Ease of Interpretation	Moderate	High	High	Variable	

This table presents a qualitative comparison synthesized from multiple systematic reviews and key studies. Descriptors such as ‘high,’ ‘moderate,’ and ‘low’ reflect general trends reported in the literature and may vary depending on specific assays, study design, and clinical context. Direct quantitative comparison across modalities is limited by heterogeneity in evaluation methods and populations.

## Data Availability

No new data were created or analyzed in this study. Data sharing is not applicable to this article.

## Abbreviations

The following abbreviations are used in this manuscript:

Ab	Antibody
Ag	Antigen
AI	Artificial Intelligence
AI-CT	Artificial Intelligence-Assisted Computed Tomography
AI-POCUS	Artificial Intelligence-Assisted Point-of-Care Ultrasound
AUC	Area Under the Curve
C	Control Line
cDNA	Complementary DNA
CE	Conformité Européenne
CGIA	Colloidal Gold Immunoassay
CLIA	Chemiluminescent Immunoassay
CNN	Convolutional Neural Network
CONAN	Cas3-Operated Nucleic Acid Detection
COVID-19	Coronavirus Disease 2019
copies/mL	Viral Genome Copies per Milliliter
CRISPR	Clustered Regularly Interspaced Short Palindromic Repeats
CRISPR-Cas	CRISPR-Associated Protein System
CT	Computed Tomography
DETECTR	DNA Endonuclease-Targeted CRISPR Trans Reporter
EDTA	Ethylenediaminetetraacetic Acid
E	Envelope Protein
EUA	Emergency Use Authorization
FET	Field-Effect Transistor
FDA	Food and Drug Administration
fg/mL	Femtogram per Milliliter
FIA	Fluorescence Immunoassay
fM	Femtomolar
IFIA	Immunofluorescence Immunoassay
IgA	Immunoglobulin A
IgG	Immunoglobulin G
IgM	Immunoglobulin M
IoMT	Internet of Medical Things
LAMP	Loop-Mediated Isothermal Amplification
LF-CGIA	Lateral Flow Colloidal Gold Immunoassay
LF-IFIA	Lateral Flow Immunofluorescence Immunoassay
LFIA	Lateral Flow Immunoassay
LOD	Limit of Detection
LSPR	Localized Surface Plasmon Resonance
LSTM	Long Short-Term Memory
M	Membrane Protein
MIFIA	Microfluidic Immunofluorescence Immunoassay
N	Nucleocapsid Protein
NAAT	Nucleic Acid Amplification Test
NEAR	Nicking Enzyme Amplification Reaction
ng/mL	Nanogram per Milliliter
NMPA	National Medical Products Administration
NR	Not Reported
NS	Nasal Swab
NPS	Nasopharyngeal Swab
ORF1ab	Open Reading Frame 1ab
OS	Oropharyngeal Swab
OTC	Over-the-Counter
PBS	Phosphate-Buffered Saline
PCR	Polymerase Chain Reaction
pM	Picomolar
POCT	Point-of-Care Testing
POCUS	Point-of-Care Ultrasound
RdRp	RNA-Dependent RNA Polymerase
RNA	Ribonucleic Acid
ROS	Reactive Oxygen Species
RSV	Respiratory Syncytial Virus
RT-LAMP	Reverse Transcription Loop-Mediated Isothermal Amplification
RT-PCR	Reverse Transcription Polymerase Chain Reaction
S	Spike Glycoprotein
SARS-CoV-2	Severe Acute Respiratory Syndrome Coronavirus 2
SGTF	S-Gene Target Failure
SHERLOCK	Specific High-Sensitivity Enzymatic Reporter Unlocking
SPR	Surface Plasmon Resonance
T	Test Line
TGA	Therapeutic Goods Administration
TRF-LFIA	Time-Resolved Fluorescence Lateral Flow Immunoassay
VaNGUARD	Variant Nucleotide Guard
VOC	Variant of Concern
WHO	World Health Organization
